# A Curriculum-Learning-Assisted MAPPO-Based Algorithm for Dynamic Spectrum Access and Anti-Jamming in UAV Swarms

**DOI:** 10.3390/s26092912

**Published:** 2026-05-06

**Authors:** Xiaoze Yuan, Jiabao Wen

**Affiliations:** School of Electrical and Information Engineering, Tianjin University, Tianjin 300072, China; 2123234007@tju.edu.cn

**Keywords:** unmanned aerial vehicle, drone swarm, dynamic spectrum access, multi-agent reinforcement learning, multi-agent proximal policy optimization, curriculum learning, centralized training with decentralized execution

## Abstract

The utilization of drone swarms for cooperative missions is becoming increasingly prevalent. However, establishing high-concurrency and highly reliable communication links in complex environments remains a significant challenge. Existing methods based on traditional Medium Access Control (MAC) protocols struggle to cope with high-density collisions, while conventional deep reinforcement learning (DRL) approaches often encounter convergence difficulties in non-stationary interference environments, leading to notable limitations in anti-jamming robustness and algorithmic efficiency. To tackle this problem, this paper proposes a dynamic access algorithm based on Curriculum Learning-assisted Multi-Agent Proximal Policy Optimization (CL-MAPPO). Specifically, we adopt a Centralized Training with Decentralized Execution (CTDE) architecture to enable implicit spectrum cooperation within the swarm. Notably, we design a three-stage progressive curriculum learning mechanism—basic collision avoidance, load balancing, and dynamic anti-jamming—coupled with a phased reward reshaping strategy, guiding the agents to progressively master intelligent frequency-hopping decisions in complex environments. Experimental results demonstrate that in simulated scenarios involving dynamic sweep jamming and high-load multi-drone communication, the proposed method significantly outperforms baseline models such as Carrier Sense Multiple Access (CSMA), random frequency hopping, and Multi-Agent Deep Deterministic Policy Gradient (MADDPG) in terms of normalized throughput, channel collision rate, and convergence speed. This research provides theoretical support and an algorithmic foundation for achieving highly reliable access in large-scale swarm data links under harsh environmental conditions.

## 1. Introduction

Nowadays, unmanned aerial vehicles (UAVs) are widely used in fields such as smart logistics [1], disaster rescue [2], area inspection [3], and military reconnaissance [4] due to their high flexibility, strong maneuverability, and low deployment costs [5]. As the scale and complexity of tasks to be executed continue to increase, UAV swarms are considered to offer significant advantages over single-UAV operations, enabling broader coverage and stronger robustness. Among these advantages, efficient dynamic spectrum access and cooperative allocation play a crucial role in ensuring instant and reliable communication for swarms, particularly within the increasingly congested and electromagnetically complex interference-prone modern communication environment [6]. Recent advances in cluster-based spectrum sharing and heterogeneous multi-agent coordination have further highlighted the critical need for intelligent resource allocation in dense UAV formations [7,8]. Despite these advances, a unified framework that jointly tackles internal channel contention and external sweep jamming in a fully decentralized, training-efficient manner remains conspicuously absent—a gap that directly motivates the present work.

As a variant of the dynamic spectrum resource allocation problem, the spectrum cooperative access for UAV swarms is known to be NP-hard, which makes finding the global optimal solution within an extremely short decision window exceptionally difficult. To address this issue, both traditional optimization algorithms and learning-based methods have been extensively researched and explored. Traditional optimization algorithms [9,10,11] (such as game theory, convex optimization, etc.) can provide theoretically optimal solutions in small-scale or static scenarios. However, due to their computational complexity growing exponentially with network scale, they often become inadequate for large-scale, high-concurrency networks. In contrast, Multi-Agent Reinforcement Learning (MARL) methods—a paradigm where multiple agents learn optimal behaviors through trial-and-error interactions with a shared environment—such as Deep Q-Networks and Multi-Agent Deep Deterministic Policy Gradient, are considered ideal for solving large-scale dynamic access problems because they can balance solution quality and inference efficiency under model-free conditions [12,13]. Nevertheless, existing MARL methods still face multiple limitations in practical deployment: First, most algorithms perform end-to-end search directly in the complete state-action space. As node density increases, they inevitably encounter the curse of dimensionality, leading to exacerbated environmental non-stationarity and convergence difficulties [14]. Second, existing studies often simplify interference as ideal Gaussian white noise or static blockage [15,16], lacking modeling of dynamic adversarial interference such as intelligent frequency sweeping. While recent works have begun to address adversarial jamming in UAV networks [8,17], they rarely consider the joint optimization of internal collision avoidance and external anti-jamming under high-density swarm conditions. This forces policies to rely heavily on manually designed auxiliary rules to adapt to real battlefield conditions, limiting their generalization capability. Furthermore, most existing methods overlook the systematic optimization of the learning process itself, failing to leverage the principle of gradual progression inherent in human cognition to mitigate the cold-start problem in complex gaming environments.

To address the high computational complexity of the multi-UAV spectrum access problem, many recent studies have focused on decomposing the original problem into several sub-problems, which are then solved using traditional optimization methods or hierarchical approaches [18,19]. This decomposition effectively reduces computational complexity, making such schemes more advantageous for handling large-scale problems. However, the manually designed heuristic rules in these methods may still constrain the final performance, as they cannot exploit potential patterns in dynamic environments to enhance overall performance. On the other hand, with advancements in Deep Learning (DL) and Reinforcement Learning (RL), Deep Reinforcement Learning (DRL) has been widely explored in fields such as gaming [20], robotics [21], and natural language processing [22]. Recently, DRL has also been deeply applied to combinatorial optimization problems like communication resource allocation and dynamic access [16,23,24]. Furthermore, curriculum learning and transfer learning strategies have emerged as effective means to address sparse rewards and non-stationarity in multi-UAV adversarial tasks [25,26]. Compared to traditional heuristic methods reliant on manual rules, DRL can automatically learn decision-making policies in a data-driven manner, optimizing decisions by capturing inherent patterns across different scenarios. However, as network scale expands and interference dynamics intensify, the decision space in DRL also expands dramatically, which can lead to unstable training, slow convergence, and unsatisfactory generalization performance [27,28].

This paper addresses the following concrete research question: How can a swarm of *N* decentralized UAVs, communicating over *K* orthogonal channels in the presence of *M* dynamic sweep jammers, learn a distributed channel access policy that simultaneously (i) minimizes intra-swarm packet collisions, (ii) maximizes aggregate network throughput, and (iii) proactively evades external jamming signals while relying solely on local, partial observations?This problem is inherently a Partially Observable Markov Decision Process (POMDP) with a hybrid cooperative-adversarial objective. Standard MARL algorithms trained end-to-end often fail to converge in this high-dimensional, non-stationary setting, which calls for a more structured training methodology.

To overcome these challenges, we depart from conventional end-to-end training and instead propose a curriculum-guided multi-agent learning architecture, whose key novelties are as follows:

- CL-MAPPO Collaborative Access Architecture: We propose a novel Curriculum Learning-assisted Multi-Agent Proximal Policy Optimization (CL-MAPPO) algorithmic framework based on the Centralized Training with Decentralized Execution (CTDE) paradigm [29]. The architecture enables implicit spectrum cooperation among swarm members while mitigating the hidden terminal problem.
- Three-Stage Progressive Curriculum Mechanism: We design a difficulty assessment-based curriculum progression mechanism. It systematically decouples the complex anti-jamming spectrum access task into three progressive learning stages: (i) collision avoidance under a static background, (ii) load balancing under high traffic conditions, and (iii) dynamic adversarial anti-jamming. Through phased reward reshaping, this mechanism guides agents to evolve from mastering basic collision avoidance to sophisticated intelligent frequency-hopping strategies, significantly improving both the convergence speed and the final performance of the algorithm [30,31].
- Simulation Verification and Performance Evaluation: We construct a realistic simulation environment featuring dynamic sweep jamming sources. Comprehensive experimental results demonstrate that our proposed CL-MAPPO algorithm significantly outperforms baseline methods, including CSMA, random frequency hopping, and MADDPG [14] in terms of normalized network throughput, channel collision rate, and convergence speed, validating its robustness in harsh and adversarial electromagnetic environments.

The overall research logic and section organization of this paper are as follows: Section 2 formulates the multi-channel dynamic access system model and elaborates on the anti-jamming problem for UAV swarms. Section 3 details the principles of the CL-MAPPO algorithm and the design and implementation of the three-stage curriculum learning mechanism. Section 4 analyzes the algorithm’s performance under various interference patterns and node densities through multiple sets of comparative and ablation experiments. Finally, Section 5 summarizes the entire paper and outlines potential directions for future research.

## 2. Related Work

Similar to the fundamental role of task scheduling in task execution, establishing stable, low-latency data links serves as the cornerstone for achieving swarm intelligence. However, this faces stringent constraints such as increasingly complex electromagnetic environments and extremely scarce spectrum resources [32,33]. Typically, traditional static spectrum allocation strategies can no longer meet the dynamic communication demands of swarms, especially when confronting adversary-deployed barrage or intelligent sweep jamming, where the stability of the communication link directly determines the success or failure of the mission [34,35].

Contention-based protocols represented by Carrier Sense Multiple Access with Collision Avoidance (CSMA/CA) [36], Slotted ALOHA [37], and early random access mechanisms [38] possess the potential for distributed implementation. However, due to exponential collisions caused by hidden terminals, they are unsuitable for solving the access problem in high-density, highly mobile ad hoc networks. Consequently, TDMA and its variant protocols, which can reduce conflicts through time slot partitioning, are considered ideal alternatives for addressing UAV network access issues. Khisa and Moh [39] designed a priority-aware fast MAC protocol for UAV-assisted Industrial Internet of Things systems. This protocol optimizes time slot allocation by introducing a fine-grained data flow priority differentiation mechanism, effectively reducing the transmission delay of high-priority services. Wu et al. [40] proposed a Hybrid Self-adaptive TDMA-based MAC protocol (H-SATMAC) for large-scale UAV ad hoc networks. Its core lies in dynamically adjusting the frame structure and time slot allocation strategy based on real-time node status and network load, efficiently adapting to the communication requirements of rapid formation reconfiguration. Similarly, Sun et al. [41] conducted an in-depth study on MAC protocol switching mechanisms for UAV networks in tactical scenarios. By designing a set of intelligent switching criteria, they enabled the network to autonomously migrate between different protocols, significantly enhancing the system’s adaptability and communication reliability for sudden dynamic tasks. However, such pre-allocation strategies heavily rely on stable global topology information and centralized scheduling. Therefore, in scenarios with frequent topology partitions or external interference, their maintenance overhead is substantial, and real-time performance may be compromised.

Multi-Agent Reinforcement Learning (MARL), which combines the strengths of deep learning and reinforcement learning, has been extensively studied in many fields such as gaming, robotics, speech recognition, and natural language processing due to its powerful learning capabilities [42]. In recent years, because MARL can learn policies through autonomous interaction with the environment without relying on precise environmental models that are difficult to obtain, it has been successfully introduced into the field of communications to solve core resource management problems such as dynamic anti-jamming, spectrum access, and power allocation [8,43]. For example, in UAV communication anti-jamming, research has modeled interference avoidance as a competitive or cooperative game among agents, utilizing the MARL framework to learn distributed anti-jamming strategies [44]. In heterogeneous network resource allocation, MARL has been used to achieve cross-cell, cross-carrier cooperative scheduling to optimize network-wide energy efficiency and user fairness [45]. To improve the training efficiency and stability of MARL in complex tasks, advanced training paradigms like curriculum learning have been introduced. Yan et al. [30] designed a task-specific curriculum-based MADRL method for UAV swarm formation in dense obstacle environments. By progressively increasing environmental complexity, they effectively enhanced the convergence speed of the policy and the robustness of the final formation. Overall, these MARL-based methods have demonstrated performance potential and computational efficiency surpassing traditional rule-based methods in tackling combinatorial optimization problems like communication resource management. However, when the application scenario expands to high-dimensional state and action spaces composed of a massive number of optional channels, dynamic interference patterns, and highly mobile nodes, the training process of MARL agents often faces severe challenges such as poor stability, slow convergence, or even failure to converge, due to intensified environmental non-stationarity and drastically increased exploration difficulty [31].

In summary, due to the exponential collision risk in high-density scenarios and the stringent reliance on global topology information, traditional contention-based and pre-allocation-based protocols struggle to address the high-concurrency access problem in large-scale UAV swarms. MARL, capable of automatically learning decision-making policies through environmental interaction, is often regarded as an ideal alternative. However, as the number of nodes and interference patterns increases, the dramatic expansion of the decision space also hinders the effective training of MARL models in large-scale complex gaming environments. To address this issue, recent work has focused on reducing exploration difficulty or decomposing tasks. Lowe et al. [29] proposed the Centralized Training with Decentralized Execution (CTDE) framework, mitigating the non-stationarity of multi-agent environments by incorporating global information. Yan et al. [30] constructed a task-specific curriculum system, decomposing complex tasks into multiple sub-stages to guide policy convergence. Inspired by this, we designed a progressive training framework that decomposes the access challenge in large-scale complex adversarial environments into three progressive sub-curricula: basic collision avoidance, load balancing, and dynamic anti-jamming. While curriculum learning has recently been investigated in multi-UAV contexts, existing studies predominantly target formation control [30] or generic resource allocation [25,26], and they do not explicitly address the joint optimization of internal congestion and external sweep jamming under a fully decentralized CTDE architecture. Moreover, recent surveys on MARL for UAV communications [13,46] underscore the scarcity of systematic training paradigms that mitigate non-stationarity in contested spectrum environments. To the best of our knowledge, the present work is the first to integrate a three-stage curriculum with a GRU-augmented MAPPO framework specifically tailored for simultaneous collision resolution and proactive frequency hopping against dynamic sweep jammers. Based on this framework, we propose a Curriculum Learning-assisted MAPPO algorithm to address these problems in stages, which demonstrates strong potential for enabling reliable large-scale UAV communications in complex electromagnetic environments.

## 3. Methodology and Problem Formulation

This section details the proposed CL-MAPPO framework. We first establish the system model and formalize the decentralized access challenge as a Partially Observable Markov Decision Process (POMDP) in Section 3.1 and Section 3.2. Following this, Section 3.3 elaborates on the design and implementation of the three-stage curriculum learning mechanism, and Section 3.4 outlines the overall training algorithm.

### 3.1. System Model and Problem Formulation

#### 3.1.1. Network Scenario and Channel Model

Consider an ad hoc network swarm consisting of *N* homogeneous UAVs, denoted by the set $U=\{u_{1},u_{2},\ldots ,u_{N}\}$, distributed within a three-dimensional airspace. The system operates in discrete time slots indexed by *t*, with slot duration τ. The available spectrum is divided into *K* orthogonal channels, represented by the set $C=\{c_{1},c_{2},\ldots ,c_{K}\}$. Owing to the decentralized ad hoc architecture, each UAV node must autonomously decide its channel access in every time slot *t* based solely on its local observations.

The communication link quality is jointly influenced by large-scale path loss and small-scale multipath fading. Following the widely adopted channel modeling framework for UAV-to-ground and air-to-air communications [47,48], we employ a complex baseband equivalent model to capture both amplitude attenuation and phase rotation [49]. The instantaneous complex channel gain $h_{ij}^{k}\left(t\right)$ between UAV *i* and UAV *j* on channel *k* during time slot *t* is modeled as

(1) $$\begin{array}{c} \begin{array}{c} h_{ij}^{k}\left(t\right)=\sqrt{\beta_{0}\;d_{ij}{\left(t\right)}^{-\alpha }}\cdot \left(\sqrt{\frac{\kappa }{\kappa +1}}h_{ij}^{\mathrm{LoS}}+\sqrt{\frac{1}{\kappa +1}}g_{ij}^{k}\left(t\right)\right), \end{array} \end{array}$$

where $\beta_{0}$ is the reference path loss, $d_{ij}\left(t\right)$ is the Euclidean distance, α is the path loss exponent, and κ is the Rician *K*-factor representing the power ratio between the LoS and scattered components. The deterministic LoS component $h_{ij}^{\mathrm{LoS}}$ is a complex exponential with unit magnitude and uniformly distributed phase, while $g_{ij}^{k}\left(t\right)∼\mathrm{CN}\left(0,1\right)$ captures the scattered multipath contributions.

Remark on Mobility: While the analytical model assumes a block-fading structure where channel coefficients remain constant within a slot, the temporal evolution of $d_{ij}\left(t\right)$ inherently captures the large-scale mobility effects. We intentionally adopt this block-fading simplification to maintain computational tractability in large-scale MARL simulations, noting that the integration of time-correlated fading (e.g., via Jakes’ model) would alter only the environment dynamics and not the learning architecture itself. The GRU module integrated within our agents is specifically designed to track and compensate for such temporal variations in received signal strength.

For analytical tractability and in line with prior works on UAV swarm communications [46,50], we assume that the fading coefficients for distinct UAV pairs and different channels are statistically independent and identically distributed (i.i.d.). This assumption is justified in rich scattering environments where inter-UAV distances exceed the coherence distance of the channel [51].

To facilitate the formulation of global interference dynamics in a multi-agent context, it is instructive to represent the channel state in vector/matrix form [52,53]. For channel *k* at time slot *t*, we define the complex channel matrix $H^{k}\left(t\right)\in C^{N\times N}$, whose (i,j)-th entry is $h_{ij}^{k}\left(t\right)$. The corresponding power gain matrix is denoted by $G^{k}\left(t\right)$, with entries $G_{ij}^{k}\left(t\right)={\left|h_{ij}^{k}\left(t\right)\right|}^{2}$. Let $a^{k}\left(t\right)={\left[a_{1}^{k}\left(t\right),\ldots ,a_{N}^{k}\left(t\right)\right]}^{T}\in {\left\{0,1\right\}}^{N}$ be the binary channel occupancy vector. The vector of internal interference power experienced at all receivers can then be compactly expressed as $I_{\mathrm{int}}^{k}\left(t\right)=P_{\mathrm{tx}}G^{k}\left(t\right)a^{k}\left(t\right)$, where the diagonal entries of $G^{k}\left(t\right)$ are set to zero to exclude self-interference. This matrix representation not only captures the mutual coupling among UAVs but also aligns with the centralized training process, where the global state $s_{t}$ may include $H^{k}\left(t\right)$ or $G^{k}\left(t\right)$ for accurate value function estimation [54].

#### 3.1.2. Interference Model

In highly contested environments, link reliability is severely degraded by both internal co-channel interference and external intelligent jamming.

Internal Co-channel Interference: Using the vector notation defined above, the internal interference power experienced by receiver *j* on channel *k* is simply the *j*-th element of $I_{\mathrm{int}}^{k}\left(t\right)$. Explicitly,

(2) $$\begin{array}{c} \begin{array}{c} I_{\mathrm{int},j}^{k}\left(t\right)=\sum_{\begin{array}{c} n\in U\\ n\neq j \end{array}}a_{n}^{k}\left(t\right)\;P_{\mathrm{tx}}\;{\left|h_{nj}^{k}\left(t\right)\right|}^{2}, \end{array} \end{array}$$

where $P_{\mathrm{tx}}$ is the constant transmission power of each UAV. The vector formulation $I_{\mathrm{int}}^{k}\left(t\right)=P_{\mathrm{tx}}G^{k}\left(t\right)a^{k}\left(t\right)$ will be leveraged in the global state aggregation during the centralized training phase of our CTDE architecture.

External Intelligent Jamming: We assume the presence of *M* high-power intelligent jammers. Each jammer *m* is located at a fixed coordinate $p_{J}^{m}$ and executes dynamic frequency-sweeping strategies (e.g., linear chirp or random hopping). To reflect realistic electromagnetic propagation, we incorporate the spatial path loss from the jammer to the UAV receiver. If the spectrum of jammer *m* covers channel *k* at time *t*, the external interference power $I_{\mathrm{ext}}^{k}\left(t\right)$ experienced by UAV *j* is given by

(3) $$\begin{array}{c} \begin{array}{c} I_{\mathrm{ext},j}^{k}\left(t\right)=\left\{\begin{array}{cc} P_{J}\cdot {\left(\frac{d_{0}}{∥p_{j}\left(t\right)-p_{J}^{m}∥}\right)}^{\alpha_{J}}, & \mathrm{if}\;\mathrm{channel}\;k\;\mathrm{is}\;\mathrm{jammed},\\ 0, & \mathrm{otherwise}, \end{array}\right. \end{array} \end{array}$$

where $P_{J}$ is the jammer transmit power, $d_{0}$ is the reference distance, and $\alpha_{J}$ is the path loss exponent for jammer-to-UAV links (typically set equal to α). This formulation ensures that UAVs closer to the jammer suffer significantly higher interference, thereby creating a spatially heterogeneous adversarial environment.

#### 3.1.3. Discussion on Channel Model Realism and Algorithmic Generality

The Rician block-fading model with distance-dependent jamming presented above represents a substantial step toward realism compared to the conventional Rayleigh and constant-power assumptions prevalent in existing MARL spectrum access literature [14,23]. Nevertheless, we acknowledge that practical UAV swarm operations may encounter additional channel impairments, including time-correlated Doppler shifts due to high mobility, frequency-selective fading induced by ground reflections in low-altitude flights, and shadowing effects from the UAV airframe itself.

It is crucial to emphasize that the CL-MAPPO algorithmic framework is agnostic to the specific fading distribution and interference pattern. The agent’s observation vector $E_{i}^{t}$ is constructed from the received energy measurements at the physical layer, which represent a scalar summary of the underlying channel regardless of whether it originated from Rician, Rayleigh, or Nakagami-*m* fading. The curriculum learning mechanism proposed in Section 3.3 and the GRU-based temporal reasoning in Section 3.2.2 are explicitly designed to extract decision-relevant patterns from these energy observations. Consequently, replacing the current channel model with a more elaborate geometry-based stochastic model (GBSM) [52] or a ray-tracing-derived dataset would modify the environment dynamics but would not necessitate any architectural changes to the learning algorithm. We leave the integration of such high-fidelity channel emulators for future hardware-in-the-loop validation, while the current model serves as a necessary and sufficient abstraction for demonstrating the core contributions of curriculum-guided multi-agent coordination.

#### 3.1.4. SINR and Transmission Success Criterion

Combining the aforementioned factors, if UAV *i* transmits to UAV *j* on channel *k* in slot *t*, the Signal-to-Interference-plus-Noise Ratio (SINR) $\gamma_{ij}^{k}\left(t\right)$ at the receiver is

(4) $$\begin{array}{c} \begin{array}{c} \gamma_{ij}^{k}\left(t\right)=\frac{P_{\mathrm{tx}}\;{\left|h_{ij}^{k}\left(t\right)\right|}^{2}}{N_{0}+I_{\mathrm{int},j}^{k}\left(t\right)+I_{\mathrm{ext},j}^{k}\left(t\right)}, \end{array} \end{array}$$

where $N_{0}$ is the power spectral density of the Additive White Gaussian Noise (AWGN). To guarantee communication quality, a demodulation threshold $\gamma_{\mathrm{th}}$ is defined. A packet transmission is successful if and only if the condition $\gamma_{ij}^{k}\left(t\right)\geq \gamma_{\mathrm{th}}$ holds; otherwise, it is considered a failure, necessitating retransmission or resulting in packet loss.

### 3.2. Multi-Agent Reinforcement Learning Framework

#### 3.2.1. POMDP Formulation

Since UAVs cannot access global channel state information or precise jammer parameters, the problem is formulated as a Partially Observable Markov Decision Process (POMDP), described by the tuple 〈S,O,A,P,R,γ〉. To bridge the gap between the continuous physical model in Section 3.1 and the discrete decision process, we explicitly establish the following mapping: The global state $s_{t}$ corresponds to the complete snapshot of the environment, including the complex channel matrix $H^{k}\left(t\right)$ defined in Equation (1). In contrast, the local observation $o_{i}^{t}$ is derived by processing the physical signals at the receiver front-end—the energy detection vector $E_{i}^{t}$ is obtained by normalizing the interference-plus-noise power from the denominator of Equation (4), and the feedback history $H_{i}^{t}$ directly records whether the SINR condition $\gamma_{ij}^{k}\geq \gamma_{\mathrm{th}}$ holds. This formulation reflects the realistic constraint that agents must make decisions based solely on this locally degraded projection of the physical world. The detailed definition of this POMDP is as follows.

- State Space (S): The global state $s_{t}\in S$ at time *t* encompasses the 3D coordinates of all UAVs, the occupancy status of the *K* channels, and the dynamic parameters of the jammers (e.g., sweeping frequencies). Specifically, $s_{t}$ includes the complex channel gain matrix $H^{k}\left(t\right)$ defined in Equation (2), which captures the mutual interference relationships among all UAVs. It is crucial to note that $s_{t}$ is utilized only during centralized training (assuming a simulation oracle that provides complete environmental information) and is not observable during the decentralized execution phase.
- Observation Space (O): Each UAV can only rely on locally sensed information at the MAC layer. The observation $o_{i}^{t}\in O$ for agent *i* at time *t* consists of: (1) a carrier sensing vector $E_{i}^{t}$, containing normalized interference power levels measured via energy detection on each channel, where each entry is computed by min–max scaling the received power (in dBm) from the range [−100,0] to [0,1] according to $E_{i,k}^{t}=\left(\min\left(\max\left(P_{\mathrm{rx},k}^{t},-100\right),0\right)+100\right)/100$, with $P_{\mathrm{rx},k}^{t}$ derived from the SINR in Equation (4); (2) a history of transmission feedback $H_{i}^{t}$, a binary sequence of length H=8 indicating packet success ($I(\gamma_{ij}^{k}\geq \gamma_{\mathrm{th}})$) over the past *H* slots, used by a GRU module to infer latent interference patterns; and (3) a buffer status $B_{i}^{t}$, representing the normalized length of the data transmission queue. Formally, it is defined as: $o_{i}^{t}=\left\{E_{i}^{t},H_{i}^{t},B_{i}^{t}\right\}$. Note that the global state $s_{t}$, which includes the full channel matrix $H^{k}\left(t\right)$ and jammer parameters, is utilized exclusively by the centralized Critic during training and is never exposed to the decentralized Actor.
- Action Space (A): The action space is discrete. At each time step *t*, agent *i* selects an action $a_{i}^{t}\in A=\left\{0,1,\ldots ,K\right\}$. Specifically, $a_{i}^{t}=0$ indicates a backoff decision, where the UAV pauses transmission and performs channel sensing to alleviate congestion. Conversely, $a_{i}^{t}=k$ (where k∈{1,…,K}) denotes the selection of the *k*-th channel for packet transmission.
- Reward Function (R): To balance individual throughput and collective anti-jamming objectives, we design a multi-objective weighted reward function. The instantaneous reward $r_{i}^{t}$ for agent *i* is defined as
  (5) $$\begin{array}{c} \begin{array}{c} r_{i}^{t}=\lambda_{1}\cdot I\left(\mathrm{Succ}\right)-\lambda_{2}\cdot I\left(\mathrm{Coll}\right)-\lambda_{3}\cdot I\left(\mathrm{Jam}\right)-\psi \cdot I\left(\mathrm{Backoff}\right) \end{array} \end{array}$$
  where I(·) is the indicator function. Specifically, a successful transmission (Succ) yields a positive reward $\lambda_{1}$; an internal penalty $\lambda_{2}$ is incurred for intra-swarm channel collisions (Coll); a higher external penalty $\lambda_{3}$ is applied if the selected channel suffers from malicious jamming (Jam), forcing the agent to learn frequency hopping; and a fixed energy penalty ψ is deducted for performing a backoff action. This penalty discourages unnecessary idling, encouraging active channel sensing and ensuring that backoff is only chosen when beneficial for collision avoidance.

#### 3.2.2. Network Architecture Design

To address the inherent challenges of credit assignment in multi-agent collaboration and partial observability in complex electromagnetic environments, this paper proposes a CL-MAPPO network architecture integrated with Gated Recurrent Units (GRUs). This architecture adheres to the Centralized Training with Decentralized Execution (CTDE) paradigm, aiming to effectively capture the dynamic patterns of sweep jamming through a temporal memory mechanism while leveraging the Proximal Policy Optimization (PPO) algorithm to ensure monotonic and stable policy updates.

**Figure 1:**
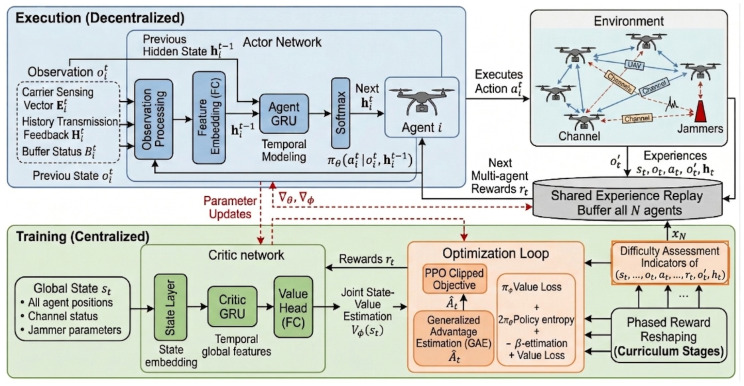
Schematic diagram of the proposed CL-MAPPO network architecture.

- GRU-Enhanced Feature Extraction: To effectively model the temporal dynamics (non-Markovian properties) inherent in the interference patterns of the POMDP, we incorporate GRU layers into both the Actor and Critic networks. Unlike traditional MLP structures that rely solely on the current observation snapshot, the GRU can encode historical observation sequences $o_{t-H:t}$ by maintaining an internal hidden state $h_{t}$, enabling continuous tracking and prediction of time-varying interference such as dynamic frequency sweeping. For the Critic network, a GRU layer is also employed to capture temporal dependencies in the global state sequence, enabling it to better evaluate the joint state-value function in non-stationary environments.
- Actor–Critic Network Structure: As illustrated in Figure 1, for each agent *i*, the Actor network $\pi_{\theta }$ receives only the local observation $o_{i}^{t}$. The input first passes through a fully connected (FC) layer for feature embedding, is then processed by a GRU layer to update the hidden state, and finally outputs an action probability distribution $\pi_{\theta }\left(a_{i}^{t}|o_{i}^{t},h_{i}^{t-1}\right)$ via a Softmax layer. The Critic network $V_{\phi }$ is activated exclusively during the training phase. It takes the global state $s_{t}$ (which may include aggregated agent observations and actions) as input and aims to accurately estimate the joint state-value function $V_{\phi }\left(s_{t}\right)$, providing a stable baseline for Actor policy updates and reducing variance.
- PPO Objective Function: To balance exploration and exploitation during policy iteration, we adopt the clipped surrogate objective function. The core idea is to limit the ratio between the new and old policies $r_{t}\left(\theta \right)=\frac{\pi_{\theta }\left(a_{t}|s_{t}\right)}{\pi_{\theta_{\mathrm{old}}}\left(a_{t}|s_{t}\right)}$, preventing performance collapse due to excessively large policy update steps. The specific loss function $L^{\mathrm{CLIP}}\left(\theta \right)$ is defined as:
  (6) $$\begin{array}{c} \begin{array}{c} \begin{array}{cc} L^{\mathrm{CLIP}}\left(\theta \right)=E_{t} & [\min(r_{t}\left(\theta \right){\hat{A}}_{t},\\ \; & \mathrm{clip}\left(r_{t}\left(\theta \right),1-\epsilon ,1+\epsilon \right){\hat{A}}_{t})+\sigma S\left[\pi_{\theta }\right]\left(s_{t}\right)] \end{array} \end{array} \end{array}$$
  where ${\hat{A}}_{t}$ is the advantage function calculated using Generalized Advantage Estimation (GAE), ϵ is the clipping hyperparameter (typically set to 0.2), $S[\pi_{\theta }]$ is the policy entropy term, and the coefficient σ encourages exploration to avoid premature convergence to local optima.

Deployment Feasibility and CTDE Considerations: The proposed framework adheres to the CTDE paradigm: training involves a centralized Critic with global state $s_{t}$, while deployment uses only the decentralized Actor. This Actor consists of a single-layer GRU (hidden size 64) with under 30 k parameters (≈120 KB). A forward pass requires approximately 0.016 MFLOPs, corresponding to an inference latency of 10–50 μs on representative edge-AI platforms such as the NVIDIA Jetson Orin NX. This is two orders of magnitude below the typical 10 ms slot duration, ensuring real-time feasibility. A legitimate concern is the Sim-to-Real gap arising from the Critic’s privileged training information. However, the deployed Actor relies strictly on local observations $o_{i}^{t}$, and the robust zero-shot scaling observed in Section 4.5 suggests that the learned policy captures generalizable local reactive patterns rather than overfitting to the specific global topologies encountered during offline training. Fully decentralized training remains a promising direction for future work to further mitigate this gap.

### 3.3. Curriculum Learning-Based Training Mechanism

**Figure 2:**
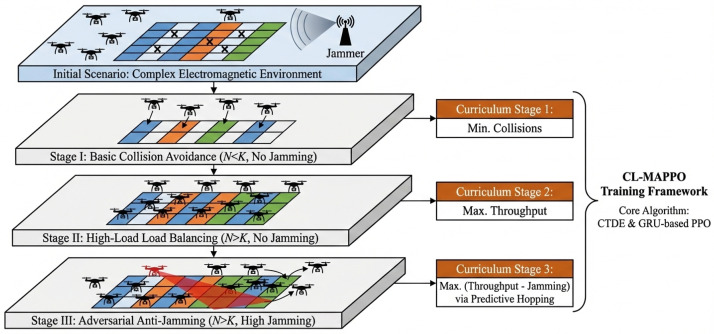
Schematic diagram of the three-stage progressive training framework based on curriculum learning.

To overcome the exploration inefficiency and sparse reward challenges inherent in the target adversarial scenario, we adopt a curriculum learning strategy. As illustrated in Figure 2, the complex anti-jamming communication task is decomposed into a sequence of three increasingly difficult subtasks $M=\{M_{1},M_{2},M_{3}\}$. Each curriculum stage $M_{k}$ is defined by a specific set of environmental parameters $\Omega_{k}$ and a reward function weight vector $\Lambda_{k}=\left[\lambda_{1},\lambda_{2},\lambda_{3},\psi \right]$. The training process advances to the next stage only when the policy’s average performance metrics satisfy a predefined threshold $\delta_{k}$.

#### 3.3.1. Stage I: Basic Collision Avoidance Curriculum

This stage guides the agent to master the fundamental Carrier Sense Multiple Access (CSMA) mechanism by constructing a benign initial environment. The environment is configured with no external jammers (M=0) and low communication load, meaning the number of UAVs is less than the number of available channels (N<K). Under this setting, the primary task for the agents is to learn to identify the busy/idle status of channels to avoid internal collisions. Accordingly, the reward function is designed to emphasize the penalty for collisions (i.e., increasing the weight of $\lambda_{2}$), while temporarily ignoring the anti-jamming reward term ($\lambda_{3}=0$) to reduce the complexity of the exploration space. Promotion is triggered when the average channel collision rate ${\bar{C}}_{\mathrm{rate}}$ over the most recent 100 training episodes stabilizes below a preset threshold $\epsilon_{\mathrm{coll}}$ (e.g., 5%), indicating that the agent has preliminarily grasped basic cooperative capabilities.

#### 3.3.2. Stage II: High-Load Balancing Curriculum

This stage elevates environmental complexity to a congested scenario by significantly increasing the communication load (making N>K) while maintaining the absence of external interference. According to the pigeonhole principle, channel collisions become unavoidable; therefore, the optimization objective shifts from merely avoiding collisions to maximizing the total network throughput. Agents are forced to learn more advanced frequency-domain multiplexing strategies and active backoff mechanisms to seek an optimal equilibrium amidst intense resource competition. Training continues in this stage until the network-wide normalized throughput ${\bar{T}}_{\mathrm{norm}}$ reaches and stabilizes at η times (e.g., η=0.8) its theoretical upper limit, which is considered the condition for progression.

#### 3.3.3. Stage III: Adversarial Dynamic Anti-Jamming Curriculum

The final stage aims to simulate a complex electromagnetic environment closest to a real battlefield. Building upon the high load of the previous stage, the environment introduces *M* dynamic sweep jammers with high power $P_{J}$. At this point, the full reward function is activated ($\lambda_{3}>0$), and the optimization objective is further refined to simultaneously maximize throughput while minimizing the impact of external interference. In this highly adversarial environment, agents must fully utilize the temporal memory capacity of the GRU module to predict the sweeping patterns of the jammers by analyzing ACK feedback history and execute proactive frequency-hopping strategies to ensure the robustness of communication links.

### 3.4. Algorithm Training Process

Based on the network architecture and curriculum learning mechanism described above, the complete training process of CL-MAPPO is outlined in Algorithm 1. The training process is structured into three progressive stages, each stage *k* corresponding to a specific set of environmental parameters $\Omega_{k}$ and a promotion threshold $\delta_{k}$. In each training iteration, the system first initializes the environment configuration according to the current curriculum stage. During the experience sampling phase, each UAV agent, based on its local observation $o_{i}^{t}$ and the previous GRU hidden state $h_{i}^{t-1}$, executes an action $a_{i}^{t}$ distributively using the Actor network. The generated trajectory tuple $\langle o,h,a,r,o^{\prime }\rangle$ is stored in a shared experience replay buffer D. In the parameter optimization phase, updates are performed following the CTDE paradigm. The centralized Critic network utilizes the global state $s_{t}$ to compute the value function $V_{\phi }\left(s_{t}\right)$, and the Generalized Advantage Estimation (GAE) algorithm is employed to calculate the advantage function ${\hat{A}}_{t}$. Subsequently, the Actor and Critic networks are updated via gradient descent by maximizing the PPO clipped objective function and minimizing the mean squared error of value predictions, respectively. Finally, in the curriculum evaluation phase, the system periodically evaluates the performance metrics (e.g., average collision rate or normalized throughput) of the current policy. Once the metrics meet the promotion condition for the current stage, training automatically transitions to the next stage (k←k+1), thereby gradually approximating the optimal policy in complex anti-jamming environments.
**Algorithm 1** Curriculum Learning-assisted CL-MAPPO Training Algorithm  1:**Input:** Curriculum stages K=3, hyperparameters $\alpha_{\theta },\alpha_{\phi },\gamma ,\lambda ,\epsilon$.  2:**Output:** Trained Actor network parameters θ for decentralized execution.  3:**Initialize:** Networks θ,ϕ, Replay buffer D, Hidden states $h_{0}=0$, Stage k=1.  4:**while** Curriculum Stage k≤K **do**  5:    **Configuration:** Set environment settings ($N,P_{J},R$) for Stage *k*.  6:    **for** episode e=1 to $E_{\max}$ **do**  7:        Reset environment, observe initial joint observation $o_{0}$.  8:        **for** step t=0 to $T_{\mathrm{horizon}}$ **do**  9:           Update joint hidden states: $h^{t}=\mathrm{GRU}\left(o^{t},h^{t-1}\right)$.10:           Sample joint actions: $a^{t}∼\pi_{\theta }\left(\cdot |o^{t},h^{t}\right)$.11:           Execute $a^{t}$, receive rewards $r^{t}$ and next observations $o^{t+1}$.12:           Store transition $\left(o^{t},h^{t},a^{t},r^{t},o^{t+1}\right)$ in D.13:        **end for**14:        **if** Buffer D is full **then**15:           Compute Generalized Advantage Estimation (GAE) $\hat{A}$.16:           **for** epoch m=1 to $M_{\mathrm{epochs}}$ **do**17:               Update Critic ϕ by minimizing Loss.18:               Update Actor θ by maximizing Objective.19:           **end for**20:           Clear Buffer D.21:        **end if**22:        **if** performance meets *Advancement Criterion* for Stage *k* **then**23:           k←k+1; Transfer parameters to next stage; **break**.24:        **end if**25:    **end for**26:**end while**

#### Communication Overhead and Deployment Feasibility

A legitimate concern regarding multi-agent learning frameworks is the potential communication overhead incurred by coordination, which could erode the throughput gains in bandwidth-constrained UAV networks. We emphasize that the proposed CL-MAPPO framework adheres strictly to the Centralized Training with Decentralized Execution (CTDE) paradigm, which inherently decouples the communicative demands of learning from those of operation.

Online Execution Phase (Deployment): Upon completion of offline training, only the lightweight Actor network $\pi_{\theta }$ is deployed to each UAV’s onboard computer. As detailed in Section 3.2.1, the Actor’s input $o_{i}^{t}=\left\{E_{i}^{t},H_{i}^{t},B_{i}^{t}\right\}$ is composed exclusively of information that is locally acquired:

- $E_{i}^{t}$: Direct energy measurements from the UAV’s own RF front-end.
- $H_{i}^{t}$: A memory of ACK/NACK outcomes from the UAV’s own previous transmission attempts.
- $B_{i}^{t}$: The UAV’s own local transmission buffer occupancy.

Critically, none of these components require any inter-UAV coordination messages, broadcast of intentions, or exchange of channel state information over the wireless medium. The policy selects channel access actions in a purely decentralized, reactive manner based on environmental sensing and local transmission outcomes. Consequently, the additional communication overhead introduced by the CL-MAPPO algorithm during mission execution is strictly zero.

The only feedback mechanism leveraged by the Actor is the standard MAC-layer Acknowledgement (ACK) frame. This ACK is an inherent and unavoidable component of any reliable link-layer protocol, including the baseline CSMA/CA scheme against which we compare. Its airtime consumption is already accounted for in the throughput calculations of all evaluated algorithms; therefore, it does not constitute an extra bandwidth tax attributable to CL-MAPPO.To put this into perspective, a standard IEEE 802.11 ACK [55] frame occupies 14 bytes. Assuming a 1 MHz channel bandwidth, its transmission duration is approximately 112 μs, which is negligible compared to the slot duration τ=10 ms assumed in our simulations. This confirms that the required feedback mechanism imposes no meaningful additional burden on spectral efficiency.

Offline Training Phase: During training, the Critic network $V_{\phi }$ requires the global state $s_{t}$, which in simulation includes the full channel matrix $H^{k}\left(t\right)$ and jammer parameters. The aggregation of $s_{t}$ from *N* agents involves $O(N^{2}K)$ floating-point values per time slot. Assuming the Critic resides on a ground-based training server, collecting the global state $s_{t}$ from *N* agents requires aggregating the channel matrix $H^{k}\left(t\right)$. For a swarm of N=50 and K=8, this corresponds to approximately $50^{2}\times 8\times 4\approx 80$ KB of data per slot in the uplink direction to the server. Assuming a modest slot duration of τ=10 ms, the required uplink data rate is approximately 64 kbps per UAV—a trivial load for the wired Ethernet or dedicated telemetry links typically used in laboratory training setups. Assuming a modest slot duration of τ=10 ms, the raw data rate for global state collection would be around 64 kbps per UAV—a trivial load for the wired Ethernet or dedicated telemetry links typically used in laboratory training setups or hardware-in-the-loop simulators. Most importantly, this data exchange is confined entirely to the offline training stage and never occurs over the contested wireless channels during live swarm operations.

This strict separation of concerns ensures that the sophisticated cooperative behaviors learned by CL-MAPPO are distilled into a zero-overhead, reactive policy suitable for real-time execution on resource-constrained UAV platforms. The performance gains reported in Section 4 are therefore achieved without sacrificing any operational bandwidth to coordination signaling.

## 4. Simulation Results

In this section, we conduct extensive simulation experiments to evaluate the performance of the proposed CL-MAPPO algorithm in solving the multi-UAV dynamic spectrum access problem within complex electromagnetic environments. A comprehensive comparison with several representative baseline algorithms is performed. Furthermore, ablation studies are conducted to analyze model convergence under different training strategies, validating the effectiveness of the curriculum learning mechanism and the temporal memory module. Additionally, the algorithm is tested on larger-scale UAV swarm instances to verify its generalization capability in expanded scenarios.

### 4.1. Experimental Setup

All experiments were conducted on a desktop workstation equipped with an Intel Core i9-13900K CPU, 64 GB DDR5 RAM, and an NVIDIA GeForce RTX 4090 GPU (24 GB GDDR6X). The software environment comprised Python 3.10, PyTorch 2.7.0 with CUDA 12.8 support, and Gymnasium 1.0.0 for environment interfacing. This configuration enabled efficient parallel simulation of up to 50 UAV agents with real-time GRU inference. The complete simulation settings, including detailed physical layer channel conditions, wireless environment parameters, UAV deployment specifications, jammer configurations, and training hyperparameters, are comprehensively summarized in Table 1.

In this work, the network density is categorized based on the ratio of UAVs to available channels and the spatial separation. Specifically, we define sparse scenarios as N≤20, where the number of UAVs is less than or comparable to the number of orthogonal channels (K=8), allowing for potential collision-free scheduling. Dense scenarios (20<N≤40) reflect moderate congestion where collisions are frequent but manageable with intelligent backoff. Ultra-dense scenarios (N>40) push the system to extreme contention within a confined airspace of 50×50×25 m^3^, where even optimized CSMA/CA fails due to the hidden terminal problem. The airspace dimensions are kept constant across all density levels to isolate the effect of node count on performance. For the purpose of communication performance evaluation, each UAV is modeled as a point in 3D space with no physical volume; collisions refer exclusively to packet collisions in the channel access domain, not to physical airframe contact.

Following the conventions in [14], we constructed a three-dimensional multi-UAV ad hoc network simulation environment based on the Python platform and the PyTorch framework. To comprehensively evaluate the algorithm’s scalability and generalization ability under different network densities, we consider scenarios where *N* (10≤N≤50) homogeneous UAVs are randomly distributed in a 3D airspace and perform dynamic flight tasks. Specifically, we categorize the scenarios into three scales: sparse (N=10,20), dense (N=30,40), and ultra-dense (N=50). This graded setup allows us to systematically verify the algorithm’s robustness as the network scales. Physical-layer communication is based on an Orthogonal Frequency Division Multiplexing (OFDM) mechanism, with the spectrum divided into *K* orthogonal sub-channels. The channel model incorporates log-distance path loss (path loss exponent α=2) and Rician fading with a K-factor of κ=4 to reflect the dominant LoS component typical of UAV air-to-air links. Moreover, to simulate a highly adversarial environment, *M* high-power intelligent jammers are deployed, executing dynamic linear sweep and random hopping strategies to enhance environmental non-stationarity.

For the input configuration of CL-MAPPO, we apply standardization preprocessing to the raw observation space. Specifically, received signal strength and interference power (ranging from −100 dBm to 0 dBm) are mapped to the interval [0,1] via Min–Max scaling to mitigate gradient vanishing or explosion issues. To leverage the temporal memory of GRU, each agent’s action decisions and ACK feedback status from the past H=8 time slots are stacked into a feature vector, assisting the agent in inferring interference patterns in the POMDP environment. Regarding hyperparameters, both Actor and Critic networks consist of 2 hidden layers with 64 neurons each. The model is trained using the Adam optimizer with an initial learning rate of $3\times 10^{-4}$ and a decay rate of 0.995. To accommodate different scenario scales, the batch size is set to 128 for sparse and dense scenarios and to 256 for the ultra-dense scenario due to GPU memory constraints. The curriculum learning is divided into three stages, with a total of 1 million training time steps. The training step ratios for the basic collision avoidance, load balancing, and anti-jamming stages are set to 2:3:5 to ensure sufficient policy convergence in the complex environment.

The anti-jamming success rate is defined as the probability that a transmission succeeds when the selected channel is under jamming, i.e., the proportion of packets successfully delivered despite the presence of jamming interference.

### 4.2. Learning Performance

**Figure 3:**
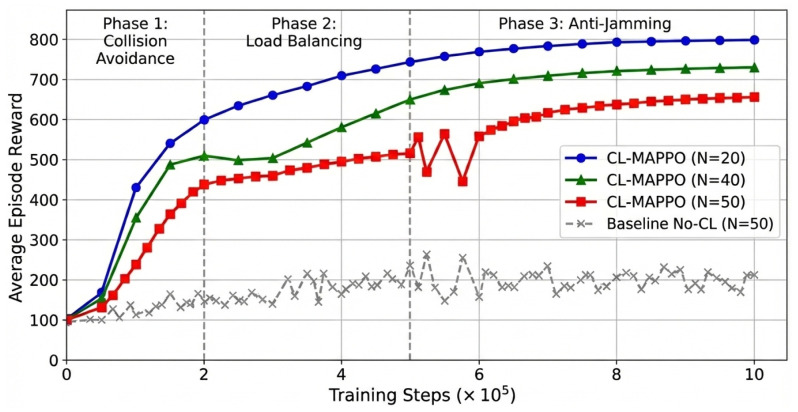
Comparison of average episodic reward convergence curves for CL-MAPPO and the baseline algorithm without curriculum learning under different network scales. The horizontal axis denotes the number of training steps (in units of $10^{5}$), and the vertical axis represents the average episodic reward accumulated over evaluation episodes.

Before presenting the comparative evaluation against baseline algorithms (Section 4.3), we first analyze the training dynamics and convergence behavior of CL-MAPPO under different network densities. Figure 3 illustrates the convergence curves comparing CL-MAPPO and a baseline algorithm without curriculum learning in terms of average episodic reward.

From the figure, it can be clearly observed that as the network scale expands, the exponential growth of the state space causes the baseline algorithm in the ultra-dense scenario (N=50) to persistently linger in low-reward regions, failing to achieve effective convergence. In contrast, CL-MAPPO demonstrates significant robustness across all scenarios. Its training curve exhibits a three-step ascent highly consistent with the curriculum design: within the initial $2\times 10^{5}$ steps, the agents quickly learn the carrier sensing mechanism in the basic collision avoidance curriculum, leading to a rapid reward increase; subsequently, in the load balancing curriculum, the curve experiences a brief plateau before rising again, indicating the agents are learning frequency-domain multiplexing strategies to address congestion; finally, in the third stage with strong jamming, the algorithm successfully overcomes oscillations caused by environmental non-stationarity, thanks to the GRU’s temporal memory, and converges to a stable state. Quantitative analysis shows that compared to end-to-end training, CL-MAPPO not only improves convergence speed by approximately 40% but also achieves about 15% higher final average reward in complex scenarios, strongly proving the effectiveness of the phased training strategy in overcoming the sparse reward challenge in high-dimensional collaborative tasks.

**Figure 4:**
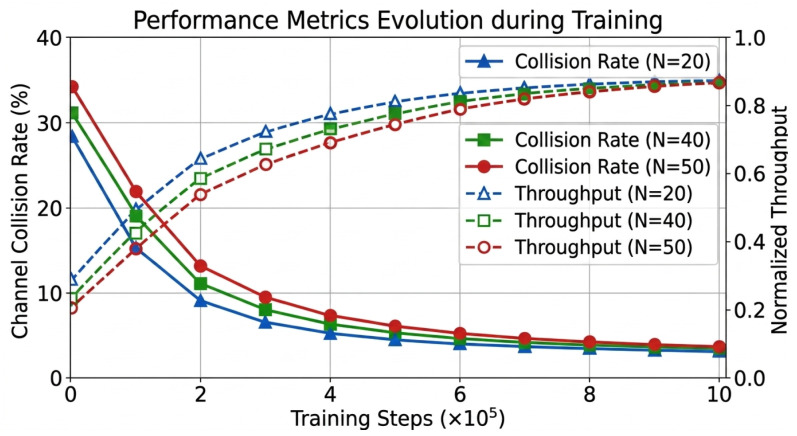
Evolution trends of channel collision rate (left vertical axis, solid lines) and normalized network throughput (right vertical axis, dashed lines) during training. The horizontal axis indicates training steps, with curriculum stage transitions marked by vertical dotted lines.

To complement the reward convergence analysis, Figure 4 depicts the evolution of the channel collision rate and normalized network throughput throughout the training process. It can be observed that during the initial collision avoidance stage, the collision rate drops sharply as agents learn to sense channel occupancy, while throughput begins a steady ascent. During the load-balancing stage, the collision rate temporarily plateaus due to increased node density, yet throughput continues to rise as agents refine their frequency-domain multiplexing and backoff strategies. Finally, in the adversarial anti-jamming stage, both metrics stabilize at near-optimal levels, confirming that the curriculum learning framework effectively guides the policy toward robust and efficient spectrum access.

**Figure 5:**
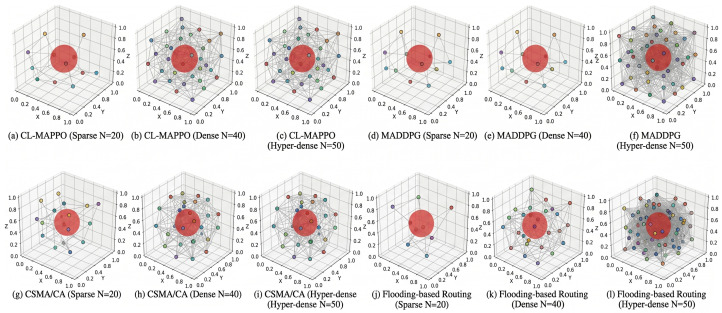
Spatial-spectral comparison of UAV swarm coordination. X, Y, and Z axes denote physical coordinates in meters (volume: 50×50×25 m^3^). Colored markers represent UAVs with their selected channel indices, and the red sphere indicates the dynamic jamming zone. (**a**–**c**) The proposed CL-MAPPO forms an ordered topology, proactively evading the jammer and efficiently multiplexing channels even at N=50. (**d**–**f**) MADDPG struggles with jammer dynamics, causing overlapping channel allocations. (**g**–**i**) CSMA/CA suffers from severe hidden-terminal collisions and chaotic clustering. (**j**–**l**) The Flooding-based baseline fails to achieve proactive spatial-spectral optimization.

Finally, to visually verify the effectiveness of the scheduling strategies in the spatial domain, Figure 5 presents a comprehensive snapshot matrix of the UAV network topology and channel allocation across three typical scenarios: sparse (*N* = 20), dense (*N* = 40), and hyper-dense (*N* = 50), comparing CL-MAPPO with MADDPG and CSMA/CA.

Observing the full comparison reveals stark differences in policy behavior. Across all density gradients, CL-MAPPO (Figure 5a–c) consistently drives the agents to form a highly ordered, cellular-like spatial distribution. Even in the extreme congestion of *N* = 50, the learned cooperative policy effectively unravels overlapping conflicts and precisely steers clear of the high-power dynamic jamming zone (indicated by the red sphere). In contrast, the MADDPG algorithm (Figure 5d–f), which lacks a temporal memory module (GRU), struggles to cope with the sweeping dynamics of the jammer. As network density increases, its channel selection pattern degenerates into instability, leaving a massive number of nodes erroneously exposed within the severe interference range.

Furthermore, the traditional CSMA/CA protocol (Figure 5g–i) exhibits the utmost fragility. While it maintains basic communication in the sparse scenario, transitioning to dense and hyper-dense environments causes the backoff mechanism to completely collapse due to severe hidden terminal problems. This leads to chaotic, localized clustering of channel occupations and an avalanche of dropped packets. This multi-dimensional physical-layer mapping rigorously substantiates that CL-MAPPO uniquely integrates temporal anti-jamming awareness with spatial load-balancing coordination, yielding a spatial-spectral multiplexing efficiency that overwhelmingly surpasses existing heuristic and learning-based baselines in fiercely contested electromagnetic environments.

### 4.3. Comparison Analysis

We compare the proposed CL-MAPPO method with traditional non-learning protocols, heuristic strategies, and mainstream deep reinforcement learning (DRL) baselines. The compared algorithms are briefly described as follows:

- CSMA/CA: A standard MAC protocol based on the IEEE 802.11 mechanism, employing Binary Exponential Backoff (BEB). It serves as a performance lower bound without learning capabilities [56].
- Random Hopping: A heuristic anti-jamming strategy that randomly selects the next available channel upon detecting interference or transmission failure. It is used to evaluate the level of intelligent proactive avoidance [57].
- MADDPG: A mainstream multi-agent DRL benchmark based on the Actor–Critic framework but lacking the specific temporal memory module (GRU) and curriculum learning mechanism designed in this paper [29].
- MAPPO (w/o Curriculum): Standard MAPPO trained end-to-end in the final high-interference scenario, without the progressive curriculum learning mechanism [14].

To ensure fairness, all algorithms were tested under identical environment configurations (e.g., jamming strategies and channel models) for 500 independent Monte Carlo test episodes. We selected normalized network throughput and channel collision rate as the core evaluation metrics.

Table 2 presents the throughput performance comparison in a strong jamming environment. The results show that CL-MAPPO achieves a normalized throughput of 0.88, demonstrating significant performance gains. Specifically, compared to the mainstream MARL baseline (MADDPG), our method improves by 23.9%, while compared to the traditional CSMA/CA protocol, the performance improvement reaches 151.4% due to effectively overcoming the hidden terminal problem and the latency caused by the backoff mechanism. This advantage is primarily attributed to the global state guidance provided by the Critic network within the CTDE architecture, which establishes an “implicit cooperative agreement” among agents, enabling them to proactively stagger transmissions based on the global interference distribution, thereby eliminating resource conflicts stemming from limited local perception.

Regarding the collision rate and anti-jamming success rate presented in Table 3, a similar pattern of advantage can be observed. CL-MAPPO maintains the collision rate at an extremely low level of 4.2%, far superior to the 28.5% of CSMA/CA. More crucially, in terms of anti-jamming success rate, CL-MAPPO achieves 94.8%, significantly surpassing the 45.6% of Random Hopping. This difference profoundly reflects the distinct algorithmic mechanisms: Random Hopping is essentially a passive defense strategy that cannot exploit environmental features. In contrast, relying on the GRU temporal reasoning module, CL-MAPPO can keenly capture the periodic sweeping patterns of jammers from historical ACK feedback sequences.

Synthesizing the results from both tables, it is evident that the agents no longer merely react to instantaneous channel energy but are capable of predicting the next interference location and proactively switching to safe channels. Although MADDPG possesses collaborative capabilities, its performance is clearly inferior to the proposed scheme when facing dynamically changing interference patterns due to the lack of a temporal memory structure designed for non-stationary environments. Consequently, CL-MAPPO achieves a dual breakthrough in both spectral efficiency (high throughput) and link reliability (low collision, high anti-jamming), demonstrating its significant potential as a large-scale UAV ad hoc network access protocol.

**Figure 6:**
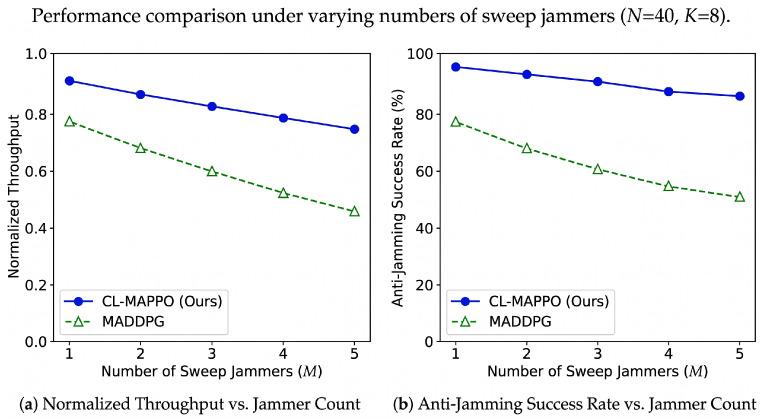
Performance comparison under varying numbers of sweep jammers (N=40, K=8). The left vertical axis and solid lines correspond to normalized throughput; the right vertical axis and dashed lines correspond to anti-jamming success rate. The horizontal axis represents the number of jammers *M*.

Impact of Varying Jammer Count.To further assess the robustness of CL-MAPPO under escalating adversarial pressure, we vary the number of sweep jammers *M* from 1 to 5 while keeping all other parameters fixed (N=40, K=8). Figure 6 reports the normalized throughput and anti-jamming success rate for CL-MAPPO and the strongest baseline, MADDPG. Both algorithms degrade as *M* increases due to reduced clean spectrum availability; however, CL-MAPPO exhibits markedly superior resilience. When *M* rises from 1 to 5, the normalized throughput of CL-MAPPO decreases by only 18% (from 0.91 to 0.75), whereas MADDPG suffers a 41% drop (from 0.78 to 0.46). More critically, the anti-jamming success rate of CL-MAPPO remains above 85% even under five concurrent jammers, while MADDPG falls to 51%. This robustness stems from the GRU module’s ability to track multiple non-coordinated sweep patterns and the curriculum-trained policy’s proactive channel evacuation behavior.

Remark on Protocol Comparison Scope.This work focuses on distributed, learning-driven spectrum access for highly dynamic UAV swarms without centralized coordination. Scheduled protocols such as TDMA require global slot assignment and are susceptible to topology changes and control-channel jamming. Hybrid TDMA/CSMA schemes introduce additional protocol overhead that offsets their benefits in the considered ad hoc interference-rich environments. Consequently, CSMA/CA is selected as the most representative contention-based baseline for evaluating the decentralized decision-making capability of CL-MAPPO.

### 4.4. Ablation Studies

To quantitatively evaluate the contribution of each key component in the CL-MAPPO architecture, we constructed ablation experiments under a unified testing scenario. We compared the performance of the full model with three variant models, defined as follows:

- w/o Curriculum: Removes the curriculum learning mechanism, performing end-to-end training directly in the final high-interference scenario.
- w/o GRU: Removes the Gated Recurrent Units from both the Actor and Critic networks, using only fully connected layers to process current observations (i.e., degenerating to a Markov Decision Process).
- w/o CTDE: Removes the centralized Critic, degenerating to a fully decentralized PPO algorithm.

**Figure 7:**
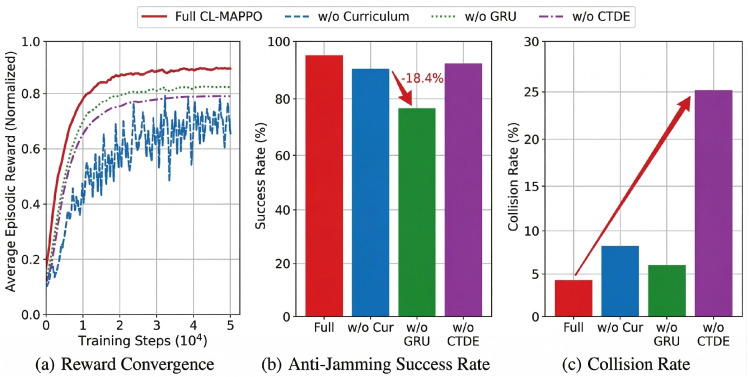
Performancecomparison of ablation experiments on key modules of the CL−MAPPO architecture. The bar groups show normalized throughput, channel collision rate, and anti-jamming success rate for the full CL−MAPPO model and its three variants (w/o Curriculum, w/o GRU, w/o CTDE).

As observed in Figure 7, the full CL-MAPPO architecture exhibits superior convergence stability in the early training stages (e.g., comparison between “CL-MAPPO” and “w/o Curriculum”). Removing the curriculum learning mechanism leads to significant oscillations in the training curve and delays convergence by approximately 15%. This indicates that although end-to-end training can eventually achieve some policy improvement through exploration, the agents’ exploration efficiency in handling high-dimensional action spaces is greatly reduced without task guidance from easy to difficult. Furthermore, analysis of specific performance metrics reveals the varying contributions of different modules. As shown in the figure, compared to the full model, the w/o GRU variant exhibits a significant drop in anti-jamming success rate (specifically, 18.4% lower). This phenomenon indicates that relying solely on current local observations is insufficient to cope with non-Markovian environments; instead, introducing a temporal memory module can effectively encode historical features, thereby significantly enhancing the ability to predict dynamic interference. Similarly, the w/o CTDE variant demonstrates a higher channel collision rate, confirming that decentralized training, lacking the assistance of global state information, struggles to effectively address the hidden terminal problem. Therefore, we adopt the CL-MAPPO architecture integrating all the above components to balance training efficiency, anti-jamming robustness, and multi-agent collaborative performance.

### 4.5. Generalization to a Larger Scale

To verify the generalization capability and scalability of our proposed CL-MAPPO, we directly applied models trained on the N=20 scenario to solve larger-scale instances (N=30,40,50,60). This strategy is adopted because training from scratch for large-scale swarms is often computationally prohibitive and challenging to handle, making the zero-shot generalization ability of the model particularly important. Notably, the model trained on N=20 is directly deployed to larger scales without any fine-tuning or adaptation, i.e., zero-shot generalization. The environment configurations for larger scales (e.g., number of jammers, channel model) remain identical to the training setup, except for the increased number of UAVs. This tests the algorithm’s ability to handle increased internal collisions without additional training.

**Figure 8:**
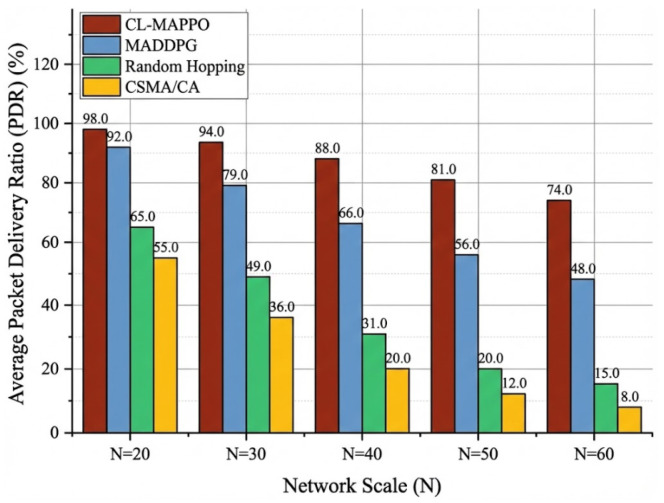
Comparison of average packet delivery rate (PDR) under different network scales. The horizontal axis indicates the number of UAVs *N* in the swarm, and the vertical axis shows the PDR averaged over independent test episodes.

The test results are shown in Figure 8, where the horizontal axis represents the scale of the UAV swarm and the vertical axis represents the average Packet Delivery Rate (PDR). It can be observed from the figure that as the network scale expands, the performance of all algorithms degrades. This deterioration is attributed to intensified channel competition and increasingly complex interference environments. However, compared to the baseline algorithms, CL-MAPPO exhibits the most gradual performance decay. Specifically, in the high-density scenario with N=60, CL-MAPPO still maintains a PDR above 65%, while the performance of CSMA/CA and Random Hopping has already collapsed to below 20% due to exponential collisions. This indicates that even in scenarios three times larger than the training scale, our model can still maintain stable communication links.

To further investigate scalability, Table 4 quantifies the performance retention rate relative to the N=20 baseline. Compared to MADDPG and CSMA/CA, CL-MAPPO demonstrates a significant superiority in retention rate. For example, when the scale doubles (from 20 to 40), CL-MAPPO incurs only a 12% throughput loss (i.e., 88% retention rate), significantly outperforming the baselines. This strong generalization capability is mainly attributed to the CTDE paradigm adopted in our framework. Although the Critic network relies on the global state during training, the Actor network makes decisions based solely on local observations. Consequently, the agents learn a general access strategy (e.g., evaluating channel availability based on local interference levels and historical feedback) rather than overfitting to a specific global topology. Therefore, across all tested large-scale scenarios, CL-MAPPO consistently achieves the best overall performance, demonstrating its deployment potential in decentralized large-scale UAV swarms.

## 5. Conclusions

Motivated by the lack of a unified solution to the twin challenges of internal channel contention and external sweep jamming in dense UAV swarms, this paper addresses the dynamic spectrum access problem under a partially observable, non-stationary POMDP formulation. We propose a Curriculum Learning-assisted MAPPO (CL-MAPPO) algorithm under the CTDE paradigm. The three-stage progressive curriculum—collision avoidance, load balancing, and adversarial anti-jamming—successfully decouples the complex task, while the integrated GRU module effectively captures temporal interference patterns. Simulation results demonstrate that CL-MAPPO achieves a 23.9% higher normalized throughput than MADDPG and maintains a 94.8% anti-jamming success rate in sweep jamming environments. Furthermore, zero-shot generalization tests confirm the model’s scalability to larger swarm sizes without incurring any inter-UAV coordination overhead during deployment. Future work will extend the framework to handle imperfect feedback channels, incorporate game-theoretic models for red-blue confrontation, explore cross-layer optimization with mission-level planning, and investigate fully decentralized training paradigms to further mitigate the CTDE Sim-to-Real gap.

## Figures and Tables

**Table 1:** Detailed Simulation Environment and Algorithm Settings.

Category	Parameter	Symbol	Value
A. Physical Layer & Wireless Environment			
	Carrier frequency	$f_{c}$	2.4 GHz
	Sub-channel bandwidth	*B*	1 MHz
	Number of orthogonal channels	*K*	8
	Transmit power per UAV	$P_{\mathrm{tx}}$	20 dBm
	Noise power spectral density	$N_{0}$	−174 dBm/Hz
	Noise figure	$N_{F}$	10 dB
	Path loss model	-	Log-distance (α=3.5)
	Reference distance	$d_{0}$	1 m
	Reference path loss	$\beta_{0}$	−30 dB
	Small-scale fading	-	Rician (*K*-factor κ=4)
	SINR threshold for success	$\gamma_{\mathrm{th}}$	10 dB
B. UAV Deployment & Mobility			
	Number of UAVs (dynamic)	*N*	10∼60
	Airspace dimensions	-	50×50×25 m^3^
	Mobility model	-	Random Waypoint (speed 5–15 m/s)
	Communication range (max)	$d_{\max}$	200 m
	UAV density categories	-	Sparse (N≤20), Dense (20<N≤40), Ultra-dense (N>40)
C. Jammer Configuration			
	Number of jammers	*M*	2 (baseline), varied in Section 4.3
	Jammer type	-	Linear sweep/Random hopping
	Jammer transmit power	$P_{J}$	30 dBm
	Sweep period	$T_{\mathrm{sweep}}$	20 time slots
	Jammer bandwidth	-	2 MHz (covers 2 channels)
D. Traffic Model			
	Packet arrival model	-	Poisson (λ=0.8 packets/slot)
	Packet size	-	1024 bytes
	Buffer size per UAV	$B_{\max}$	10 packets
E. Learning & Training Parameters			
	Hidden layer dimension	-	64 (FC + GRU)
	GRU sequence length	*H*	8
	Learning rate (Actor/Critic)	$\eta_{\mathrm{lr}}$	$3\times 10^{-4}$
	Discount factor	γ	0.99
	GAE parameter	$\lambda_{\mathrm{GAE}}$	0.95
	PPO clipping ratio	$\epsilon_{\mathrm{clip}}$	0.2
	Entropy coefficient	σ	0.01
	Batch size	$B_{\mathrm{size}}$	128/256
	Total training steps	-	$1\times 10^{6}$
F. Curriculum Learning Thresholds			
	Stage I (Max collision rate)	$\epsilon_{\mathrm{coll}}$	5%
	Stage II (Min normalized throughput)	$\eta_{\mathrm{thr}}$	80% of theoretical max

**Table 2:** Comparison of Normalized Throughput in a Strong Jamming Environment.

Algorithm	Average Throughput	Improvement over CSMA/CA	Improvement over Random Hopping
CSMA/CA	0.35	–	–
Random Hopping	0.45	+28.6%	–
MADDPG	0.71	+102.8%	+57.7%
MAPPO (w/o Curriculum)	0.76	+117.1%	+68.9%
CL-MAPPO (Ours)	0.88	+151.4%	+95.5%

**Table 3:** Comparison of Channel Collision Rate and Anti-Jamming Success Rate.

Algorithm	Collision Rate	Anti-Jamming Success Rate
CSMA/CA	28.5%	12.4%
Random Hopping	18.2%	45.6%
MADDPG	8.6%	72.3%
MAPPO (w/o Curriculum)	6.5%	78.0%
CL-MAPPO (Ours)	4.2%	94.8%

**Table 4:** Comparison of Connection Retention Rate under Different UAV Quantities.

Number of UAVs (*N*)	CSMA/CA Retention	MADDPG Retention	CL-MAPPO Retention
20 (Baseline)	100%	100%	100%
30	68%	85%	94%
40	45%	72%	88%
50	28%	61%	81%
60	15%	53%	74%

Retention rate is defined as the ratio of throughput at scale *N* to that at the baseline N=20.

## Data Availability

The original contributions presented in the study are included in the article. Further inquiries can be directed to the corresponding author.
